# Diagnosis and Management of Interstitial Lung Disease in Patients with Connective Tissue Diseases

**DOI:** 10.1155/2021/6677353

**Published:** 2021-04-24

**Authors:** Matthew Koslow, Mehrnaz Maleki-Fischbach, Rebecca C. Keith

**Affiliations:** ^1^Division of Pulmonary, Critical Care and Sleep Medicine, Interstitial Lung Disease Program, National Jewish Health, Denver, CO, USA; ^2^Division of Rheumatology, National Jewish Health, Denver, CO, USA

## Abstract

Interstitial lung disease (ILD) associated with connective tissue diseases (CTDs) is highly heterogeneous in its clinical presentation and course. The diagnosis and management of CTD-ILD require a multidisciplinary approach involving, at minimum, a rheumatologist, a pulmonologist, and a radiologist. Close monitoring of patients with CTD-ILD is important to enable early detection of disease progression and inform decisions regarding the initiation or escalation of pharmacotherapy. In the absence of guidelines regarding how CTD-ILDs should be treated, clinicians face difficult decisions on when to use immunosuppressant and anti-fibrotic therapies. The importance of a multidisciplinary and individualized approach to the diagnosis and management of CTD-ILD is highlighted in the three case studies that we describe in this article.

## 1. Introduction

Interstitial lung disease (ILD) can develop as a serious manifestation of connective tissue diseases (CTDs) that leads to increased morbidity and mortality [1–5]. The clinical course of CTD-ILD is variable and unpredictable. Some patients with fibrosing CTD-ILDs develop a progressive course characterized by increasing fibrotic abnormalities on high-resolution computed tomography (HRCT), decline in lung function, worsening symptoms, and early mortality [2, 5, 6]. Immunosuppression is the mainstay of therapy for CTDs, but evidence for the effectiveness of immunosuppression in slowing the progression of fibrosing CTD-ILD is limited. Recently, based on the results of randomized placebo-controlled trials [7, 8], the tyrosine kinase inhibitor nintedanib became the first drug to be approved by the FDA for slowing decline in forced vital capacity (FVC) in patients with ILD associated with systemic sclerosis and for the treatment of chronic fibrosing ILDs with a progressive phenotype. In the absence of guidelines or clinical consensus regarding how CTD-ILDs should be treated, clinicians face a challenge in deciding when and how to use immunosuppressant and anti-fibrotic therapies. Here we present three case studies to illustrate the importance of taking a multidisciplinary and individualized approach to the diagnosis and management of CTD-ILDs.

## 2. Case 1: A 50-Year-Old Woman with a Four-Year History of Cough

A 50-year-old woman presented with a four-year history of cough. She was a lifelong non-smoker and worked as an administrative assistant, and her hobbies included embroidery. In the four years prior to presentation, she had received several courses of antibiotics for acute and recurrent bronchitis. Treatment for allergic rhinitis, asthma, and gastroesophageal reflux did not relieve her cough, and she was referred for further evaluation.

Additional review revealed Raynaud's phenomenon. Lung auscultation was notable for inspiratory crackles over the posterior lower lung zones bilaterally. Her fingertips were rough, cracked, and fissured. Nailfold capillaroscopy identified significant capillary tortuosity with a few dilated loops without capillary dropout or hemorrhage.

Pulmonary function tests demonstrated a restrictive pattern, with a total lung capacity (TLC) of 3.91 L (79% predicted), forced expiratory volume in one second (FEV_1_) of 1.82 L (64% predicted), and FVC of 2.21 L (65% predicted). Her FEV_1_/FVC ratio was 80, without significant bronchodilator response. Diffusion capacity of the lung for carbon monoxide (DLco) was markedly reduced at 12.5 mL/min/mmHg (48% predicted). Oxygen titration demonstrated desaturation from 94% at rest to 87% within 4 minutes of walk.

Inflammatory markers erythrocyte sedimentation rate (ESR) and C-reactive protein (CRP) were elevated at 45 mg/L (normal value: <30 mg/L) and 1.5 mg/dL (normal value: <0.4 mg/dL), respectively. Additional results included antinuclear antibody (ANA) titer 1 : 40 and negative results for rheumatoid factor (RF), anti-cyclic citrullinated peptide (anti-CCP) antibody, anti-myeloperoxidase (MPO) antibodies, anti-proteinase-3 (PR3) antibody, anticentromere antibody, anti-Scl-70 antibody, anti-Sjögren's syndrome A (SSA) and Sjögren's syndrome B (SSB) antibodies, anti-Jo-1 antibodies, creatine phosphokinase (CPK), and aldolase.

**Figure 1:**
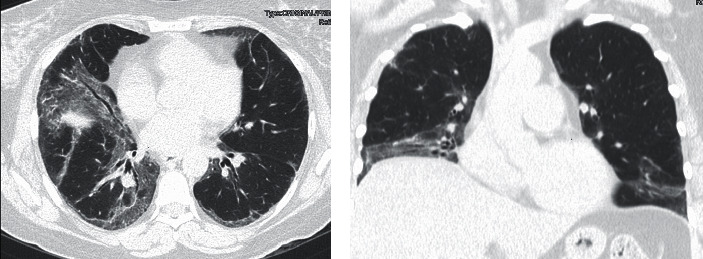
High-resolution computed tomography of the chest (Case 1). (a) Confluent ground glass opacity and consolidation, mainly in the mid and lower lungs, associated with moderate traction bronchiectasis. The distribution is partially subpleural with extension along the bronchial vascular bundles. Lung volumes are markedly reduced. (b) Coronal view demonstrating peri-diaphragmatic consolidation with an abrupt transition to normal parenchyma.

**Figure 2:**
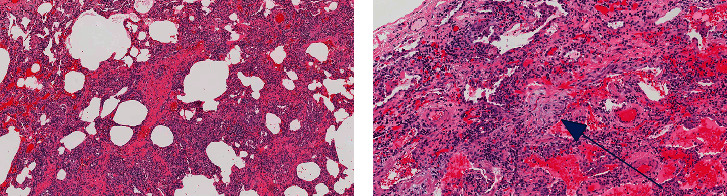
Surgical biopsy of the right lung (Case 1) demonstrates features of mixed cellular-fibrotic non-specific interstitial pneumonia (a) with scattered foci of organizing pneumonia (arrow) (b).

An HRCT scan of the chest demonstrated extensive ground glass abnormality with traction bronchiectasis, in a distribution highly suggestive of non-specific interstitial pneumonia (NSIP) (Figure 1). Prior to referral to tertiary care, the patient had undergone surgical lung biopsy, which demonstrated a mixed cellular/fibrotic pattern along with scattered foci of organizing pneumonia (Figure 2).

After a multidisciplinary discussion including pulmonary, rheumatology, and radiology specialties, a consensus diagnosis of myositis spectrum disease, more specifically anti-synthetase syndrome, was made based on the radiographic pattern of NSIP, the presence of Raynaud's phenomenon, and subtle skin abnormalities suggestive of palmar hyperkeratosis (mechanic's hands). The most common anti-synthetase antibody (anti-Jo-1) was negative, but an extended myositis panel (Oklahoma Medical Research Foundation; https://omrf.org/research-faculty/core-facilities/myositis-testing/) revealed positivity for PL-7 antibody.

Immunosuppressive therapy was initiated with prednisone and mycophenolate mofetil, which was titrated to 3000 mg daily, and Bactrim DS for prophylaxis against pneumocystis pneumonia infection. Although the patient's skin changes (mechanic's hands) and cough improved, pulmonary physiology remained stable while prednisone was tapered off. Two years later, she developed extreme fatigue and myalgia associated with elevated CPK and aldolase levels indicative of myositis. Her muscle enzymes remained elevated despite reintroduction of high-dose prednisone (1 mg/kg per day). Intravenous immunoglobulin (IVIG) infusions (2 g/kg per month) over three consecutive days for three months were initiated and led to a significant reduction in CPK and aldolase levels and improved muscle function, allowing tapering of prednisone to 5 mg per day. We note that alternative immunosuppressive therapies, such as rituximab, might have been indicated if decline in pulmonary function or myopathy was refractory to IVIG. The patient's condition remained stable on mycophenolate mofetil 3000 mg daily and she increased physical activity with pulmonary rehabilitation. We continue to monitor her condition clinically and with pulmonary function testing at 3-month intervals.

## 3. Case 2: A 52-Year-Old Man with Progressive Dyspnea

**Figure 3:**
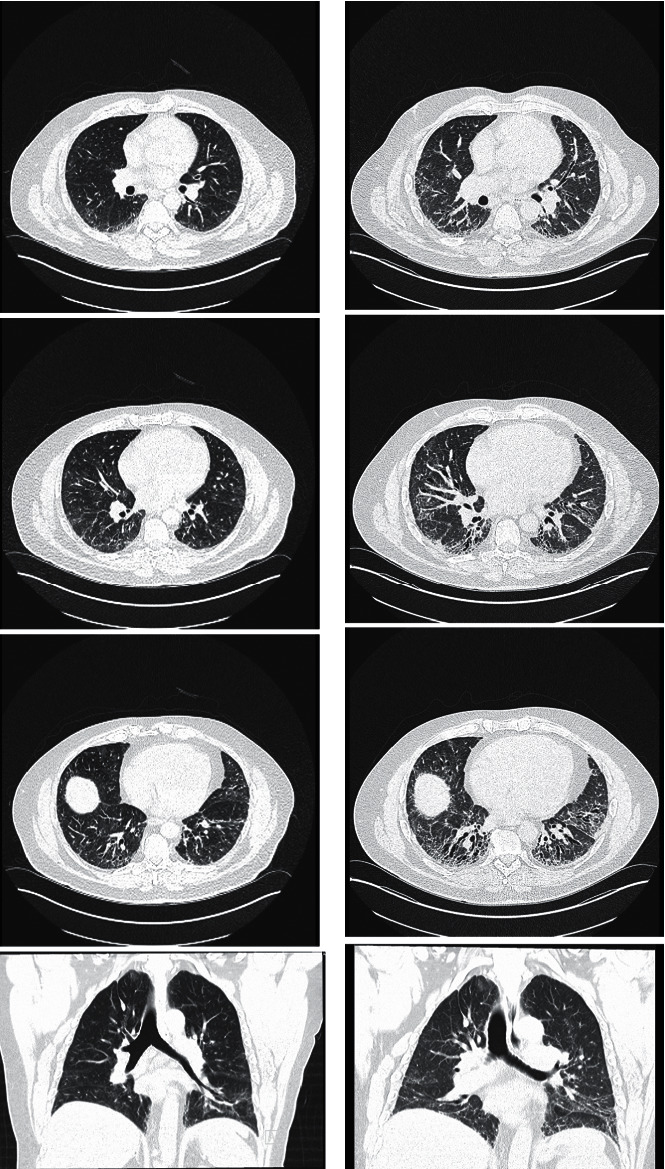
High-resolution computed tomography of the chest (Case 2). The scan 10 months prior to our evaluation (a) demonstrated a typical appearance of non-specific interstitial pneumonia (NSIP) with increased reticulation, traction bronchiectasis, lobar volume loss, and ground glass opacification in the lower lung zones. Bibasilar ground glass opacities that spare the subpleural region are characteristic for NSIP. The scan taken at our center 10 months later (b) demonstrated significant progression in fibrotic abnormalities.

A 52-year-old man presented for evaluation of progressive dyspnea. His medical history included gastroesophageal reflux disease, Hashimoto's thyroiditis, coronary artery disease, hyperlipidemia, and obstructive sleep apnea. He first noted shortness of breath approximately 1 year prior to presentation at our center. He identified shortness of breath with exertion, associated with a dry non-productive cough, as well as swelling of his ankles, severe fatigue, nail clubbing, Raynaud's phenomenon, dysphasia, and severe gastroesophageal reflux. He was referred to a local pulmonologist and underwent an HRCT scan of the chest, which demonstrated an NSIP pattern (Figure 3). He was referred to a local rheumatologist, who ultimately diagnosed scleroderma sine, based on his limited skin findings and positivity for anti-Scl-70 antibody. He was started on mycophenolate 3000 mg daily 8 months prior to presentation at our center. His dyspnea continued to worsen and he self-referred to tertiary care for further evaluation.

On physical exam, the patient had inspiratory crackles in the lower third of the lung fields bilaterally. His joint examination was notable for full range of motion. He had tenderness to palpation over his shoulders bilaterally, as well as on his metacarpophalangeal (MCP) joints and proximal interphalangeal (PIP) joints on both hands. His lower extremities demonstrated 2+ pitting edema to the midshin. His skin was notable for rare telangiectasia on his lips and chest. He had no evidence of sclerodactyly or skin sclerosis. Nailfold capillaroscopy identified multiple dilated loops with dropout or hemorrhages.

Laboratory studies were notable for positivity for ANA at 1 : 1280 homogenous and 1 : 1280 speckled. He was positive for anti-Scl-70 antibody at 73.59. Anti-RNA polymerase III, anti-double-stranded DNA and anti-extractable nuclear antigen (ENA) antibodies, and an extended myositis panel were negative. An HRCT scan demonstrated significant progression in fibrotic abnormalities compared to the HRCT scan taken 10 months previously, with a typical imaging appearance of NSIP (Figure 3). Pulmonary physiology revealed a restrictive pattern with a TLC of 3.79 L (52% predicted), FVC of 2.61 L (54% predicted), FEV_1_ of 2.47 L (65% predicted), and DLco of 10.23 mL/min/mmHg (33% predicted). This represented a 560 mL decline in FVC over 5 months while on mycophenolate. Echocardiogram did not reveal evidence of pulmonary hypertension with a right ventricular systolic pressure estimated at 27 mmHg. He had resting hypoxemia and required 2 L of supplemental oxygen at rest and 4 L of supplemental oxygen with exertion. After a multidisciplinary discussion including pulmonary, rheumatology, and radiology specialties, the consensus diagnosis was rapidly progressive fibrotic interstitial pneumonia in the context of scleroderma sine, associated with gastroesophageal reflux and mild esophageal dysmotility. Given his rapid decline, we decided to maximize immunomodulatory therapy and add anti-fibrotic therapy. Thus, rituximab was added to his mycophenolate to target inflammatory drivers of progressive lung disease and nintedanib was added to target fibrotic drivers of progressive fibrosing lung disease. Given the rapidly progressive nature of his lung disease, the patient was referred for pulmonary rehabilitation and lung transplant evaluation. He successfully underwent lung transplantation and at the time of writing, his disease had stabilized.

## 4. Case 3: A 26-Year-Old Woman in Military Service

A 26-year-old woman presented with a six-year history of dry eyes and dry mouth, shortness of breath on exertion, and fatigue. She was in active military service at the time. Prior to the onset of shortness of breath, she had been in excellent physical health. Six years earlier, she had woken up with severe conjunctival injection attributed to a corneal abrasion. She was evaluated by an ophthalmologist and Schirmer's test was positive bilaterally. She received a presumptive diagnosis of primary Sjögren's syndrome confirmed by minor salivary gland and lip biopsy. She was treated with pilocarpine eyedrops for keratoconjunctivitis sicca. A year prior to visiting our center, she developed recurrent episodes of bronchitis complicated with pneumococcal pneumonia and sepsis. Since then, she had persistent shortness of breath. Two-dimensional echocardiogram did not show pulmonary hypertension. She had mild heartburn and an esophagram showed moderate esophageal dysmotility. No evidence of aspiration was found on a tailored barium swallow. She denied having Raynaud's phenomenon.

Pulmonary function tests demonstrated largely normal lung volumes with a TLC of 4.58 (95% predicted), reduced FEV_1_ of 2.39 L (77% predicted) and FVC of 2.89 L (80% predicted), and preserved FEV_1_/FVC ratio of 95% without significant bronchodilator response. DLco was reduced at 17.74 mL/min/mmHg (62% predicted). Serum protein electrophoresis and immunofixation did not demonstrate IgG kappa monoclonal protein.

Laboratory abnormalities included a very high titer ANA 1 : 5120 speckled pattern, elevated ENA profile at 121 units (normal value: <20 units), SSA/Ro 60 at 114 units, SSA/Ro 52 at 120 units (normal value: <20 units), SSB/La at 148 units (normal value: <20 units), and RF at 20 IUs/mL. Immunoglobulin A (IgA) and IgG were elevated at 1104 mg/dL (normal range: 50–462 mg/dL) and 3956 mg/dL (normal range: 700–1620 mg/dL), respectively, while IgM and IgG were within the normal limits. C4 was decreased at 18.3 mg/dL (normal range: 19–52 mg/dL). She had mild normocytic normochromic anemia, hemoglobin of 12.1 g/dL and hematocrit of 36%, with elevated inflammatory markers erythrocyte sedimentation rate at 69 mm/hr and CRP within normal limits.

**Figure 4:**
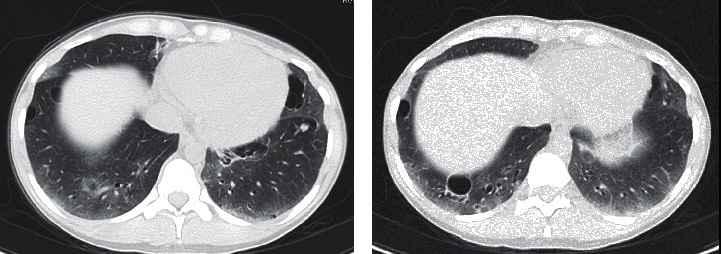
High-resolution computed tomography of the chest (Case 3). The scan prior to our evaluation (a) demonstrated pulmonary nodules, thin-walled cysts, basilar predominant ground glass attenuation, and subpleural consolidation consistent with lymphocytic interstitial pneumonia (LIP) in the setting of Sjögren's syndrome. A repeat scan (b) demonstrated increased ground glass attenuation and stable thick-walled cysts, and a few of the bilateral solid pulmonary nodules had slightly increased in size.

An HRCT scan of the chest prior to our evaluation demonstrated pulmonary nodules, thin-walled cysts, basilar predominant ground glass attenuation, and subpleural consolidation (Figure 4(a)). Repeat HRCT demonstrated increased ground glass attenuation, bilateral solid pulmonary nodules, a few of which had slightly increased in size, and stable thin-walled cysts. The pattern was consistent with lymphocytic interstitial pneumonia (LIP) in the setting of Sjögren's syndrome (Figure 4(b)). Amyloidosis and lymphoproliferative disorder/lymphoma remained additional considerations for the solid nodules, which had increased in size.

**Figure 5:**
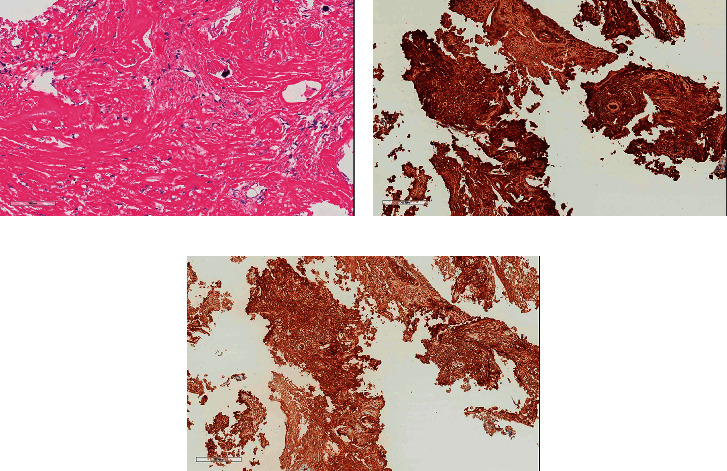
Computed tomography- (CT-) guided lung biopsy (Case 3). Hematoxylin and eosin stain (a) demonstrates amorphic eosinophilic material reminiscent of an amyloid process, whereas both kappa staining (b) and lambda staining (c) demonstrate mixed light chain deposition.

The patient underwent CT-guided lung biopsy of the right lung upper lobe. This was unremarkable for malignancy; however, immunohistochemistry was consistent with light chain amyloid, while the kappa light chain staining was the strongest (Figure 5). There was significant lambda staining, but a clear monoclonal gammopathy was not identified. Amyloid staining did not confirm amyloidosis, and a Congo red stain was not detected.

The patient was initially treated with corticosteroid taper. She complained of symptoms consistent with neuropathy as well as constant vertiginous symptoms and visual disturbances of diplopia and intermittent pressure headache. Electromyography (EMG) and nerve conductive velocity (NCV) studies were within normal limits. She was found to have acetylcholine receptor antibodies.

The patient remained an active-duty soldier. We treated her for LIP with rituximab 1000 mg, 2 doses, 14 days apart every 6 months. Later, IVIG was added to her regimen to address nervous system involvement. Her exercise tolerance improved and DLco normalized (101% predicted).

In LIP, lymphocyte and plasma cells are diffusely infiltrated in the lung parenchyma, along with polyclonal plasma cells and lymphocytes. Elevated immunoglobulins IgA and IgG can be the results of polyclonal B cells. On three occasions, the patient's serum protein electrophoresis showed polyclonal gammopathy and on one occasion it showed monoclonal gammopathy. These gammopathies resolved with rituximab therapy, as did the level of immunoglobulins and RF and sedimentation rate. Follow-up HRCT showed an overall improvement in lung involvement characterized by a decrease in ground glass opacities and the solid component of nodules with stability of cysts. We could not exclude that the patient had a low-grade pulmonary lymphoma that responded to rituximab.

## 5. Discussion

CTD-ILD is highly heterogeneous in its clinical presentation. ILD may develop after diagnosis of a CTD or may be the initial manifestation of a CTD. A diagnosis of CTD depends on careful evaluation of serologic tests in conjunction with assessment of clinical features. The gold standard for detection of CTD-ILD is an HRCT scan. The clinical course of CTD-ILDs is variable and requires close monitoring. Here we have presented three case reports that highlight the importance of thorough multidisciplinary review of clinical, laboratory, and radiologic data in the diagnosis and management of CTD-ILD.

The patient in Case 1 was diagnosed with anti-synthetase syndrome based on positivity for PL-7 antibody, despite being negative for anti-Jo-1 antibody, the most commonly detected anti-synthetase antibody [9]. Patients with anti-synthetase antibodies other than anti-Jo-1 antibody, such as anti-PL-7 and anti-PL-12, are more likely to have ILD and predominant respiratory symptoms and less likely to have muscle weakness and joint involvement than patients positive for anti-Jo-1 antibody [10, 11]. The most common patterns on HRCT in patients with anti-synthetase syndrome are NSIP or NSIP with organizing pneumonia [2, 10, 12]. Peri-diaphragmatic consolidation with an abrupt transition to normal parenchyma is highly suggestive of myositis [13, 14]. Pulmonary function testing may demonstrate a “complex restrictive” pattern in which there is a disproportionate decrease in FVC relative to TLC [15]. In Case 1, the combination of clinical, radiologic, and physiologic findings led to extended antibody testing and so to a diagnosis of anti-synthetase syndrome. The importance of screening for other manifestations of CTD, including pulmonary hypertension, gastroesophageal reflux disease, coronary artery disease, peripheral vascular disease, and vasculopathy, is also emphasized.

The underlying pathology of CTD-ILDs may be predominantly inflammatory, predominantly fibrotic, or a combination of both, and this may change as the disease progresses [16]. This may have important implications for treatment. The patient in Case 2, who had severe and rapidly progressive fibrotic interstitial pneumonia associated with scleroderma sine, had both inflammatory and fibrotic drivers of ILD, indicating that targeted treatment of both inflammation and fibrosis was required (hence the use of rituximab, mycophenolate, and nintedanib). Patient preferences are also an important consideration, as illustrated by the patient in Case 3, who was in active military service, and for whom initial treatment with mycophenolate and other immunosuppressants was considered inappropriate given the need for regular laboratory testing and the increased risk of infection [17]. Although guidelines are lacking for prophylaxis against pneumocystis pneumonia infection in patients with CTD-ILDs, risk factors for this infection may include prolonged corticosteroid use [18] and lymphopenia [19] and treatment with prednisone >20 mg daily or prednisone in combination with other immunosuppressants such as cyclophosphamide [20].

Given the unpredictable course of CTD-ILD and the need to take action promptly if ILD progresses, close monitoring of patients with CTD-ILD is important. The patients described in these case studies were monitored for progression with pulmonary function tests at 3- to 6-month intervals. Repeat HRCT scans may also reveal increases in fibrotic abnormalities, as demonstrated by Cases 2 and 3. Importantly, although a usual interstitial pneumonia (UIP) pattern on HRCT has been associated with a worse prognosis in patients with ILDs, as demonstrated in these case studies, as well as in other clinical studies [21, 22], patients with other fibrotic patterns on HRCT may also experience rapid disease progression and require regular monitoring. Close monitoring allows early detection of disease progression and changes to pharmacotherapy, ideally based on multidisciplinary discussion involving at minimum a rheumatologist, a pulmonologist, and a radiologist.

In summary, these case reports highlight the importance of taking a comprehensive multidisciplinary and individualized approach to diagnosing and managing ILD in patients with CTD. The results of pulmonary function tests and HRCT scans should be considered in addition to careful evaluation of serologic tests.

## Data Availability

Additional anonymized data on these case studies may be available on request. Please contact the authors.
